# Analysing the Effect of Energy Intensity on Carbon Emission Reduction in Beijing

**DOI:** 10.3390/ijerph20021379

**Published:** 2023-01-12

**Authors:** Gen Li, Shihong Zeng, Tengfei Li, Qiao Peng, Muhammad Irfan

**Affiliations:** 1 College of Economics & Management, Beijing University of Technology, Beijing 100124, China; 2 Faculty of Environmental Engineering, The University of Kitakyushu, Kitakyushu 808-0135, Japan; 3 Beijing Modern Manufacturing Development Research Base of Beijing Philosophy and Social Sciences, Beijing University of Technology, Beijing 100124, China; 4 Group of Information Technology, Analytics & Operations, Queen’s University Belfast, Belfast BT9 5EE, UK; 5 School of Management and Economics, Beijing Institute of Technology, Beijing 100081, China; 6 Center for Energy and Environmental Policy Research, Beijing Institute of Technology, Beijing 100081, China; 7 Department of Business Administration, Ilma University, Karachi 75190, Pakistan

**Keywords:** Beijing, energy intensity, carbon emissions, LMDI, spatial spill over effect

## Abstract

Beijing has experienced rapid economic development since the reforms and opening up. However, the traditional development model based on excessive energy consumption has posed great challenges to the ecological environment. To curb environmental degradation and achieve sustainable social development, Beijing has proposed to achieve carbon neutrality by 2050. As an important indicator of energy consumption, it is necessary to clarify how energy intensity (EI) affects carbon emissions (CE) to achieve carbon neutrality in Beijing by 2050. This study first decomposes the drivers of CE in Beijing from 2010 to 2020 using the logarithmic mean Divisia index (LMDI) method and comparatively analyses the impact of EI on CE. Then, the spatial Dubin model (SDM) is used to analyse the spatial spillover effect of EI on CE at the regional level. Finally, the macro moderating role of economic development in the effect of EI on CE is analysed. The results show that the effect of EI has been the main driver of CE reduction in Beijing. Among the industrial sectors, manufacturing and transportation have had the greatest success in reducing CE through EI reduction. At the regional level, there is a spatial spillover effect of EI on CE, and the effect of carbon reduction through the spillover effect of EI is greater than the direct effect of EI. Economic factors have an enhanced moderating effect on the process of EI affecting CE, and this moderating effect has threshold properties.

## 1. Introduction

Climate change is a major challenge for the sustainable development of human societies [1,2,3,4], and carbon emissions (CE) are the main cause of climate change [5,6,7,8,9]. As the world’s largest CO_2_ emitter [10], the Chinese government has been actively responding to climate change and has made a number of commitments to reduce CE [11,12,13]. For example, an action target was announced at the United Nations General Assembly in September 2020 to “reach the peak by 2030 and realize carbon neutral by 2060 (Double Carbon Target)”. To achieve this goal, reducing emissions in each region will play a crucial role [14].

**Figure 1:**
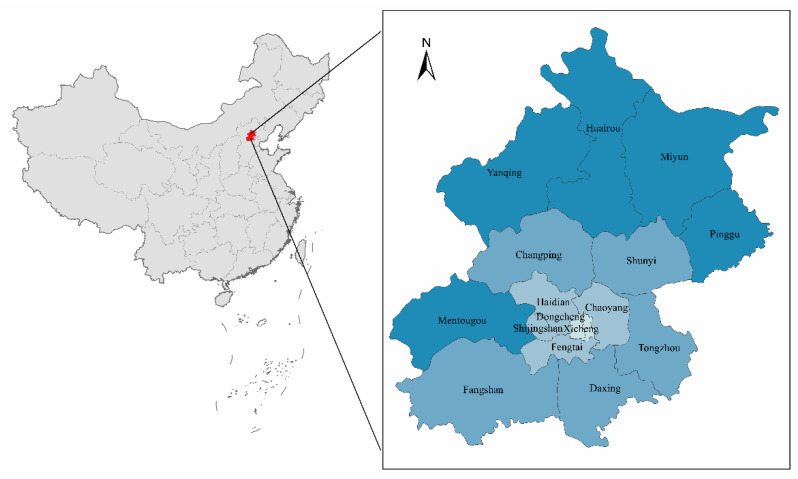
The spatial location of Beijing and its administrative division.

Beijing is located in the north-east of China, bordering the city of Tianjin to the east and Hebei province to the west (Figure 1). As the political, economic, and cultural centre of China, Beijing is far ahead in terms of economic and social development [11]. In 2021, Beijing’s gross domestic product (GDP) per capita is RMB 183,980, ranking first in China. However, the rapid economic development hides high energy consumption. According to the Beijing Statistical Yearbook, total energy consumption in Beijing has increased from 41.44 million tonnes of standard coal in 2000 to 67.621 million tonnes of standard coal in 2020. The considerable energy consumption has led to serious problems, such as environmental degradation, while promoting rapid economic development [15]. In recent years, Beijing has promulgated and implemented a number of measures to reduce emissions. In 2020, Beijing’s CE amounted to 49.6 million tonnes, a decrease of 8.94% from 2000. Despite the remarkable achievements in reducing CE in Beijing, the city is still under great pressure to reduce emissions at this stage. As the centre of scientific innovation and international exchange in China, Beijing is also responsible for achieving the “Double Carbon” goal and providing incentives and examples for other cities to make a low-carbon transition [16]. Therefore, it is essential to identify the main drivers of CE in Beijing and take targeted policy measures.

Excessive energy consumption is the main cause of CE [17] and improving energy efficiency is critical to reducing CE. As a measure of the overall efficiency of energy use, energy intensity (EI) can reflect the degree of economic dependence on energy. Reducing EI helps to decouple energy consumption from economic development and is therefore considered an effective pathway to green development [18,19]. Therefore, understanding the relationship between EI and CE in Beijing is conducive to the formulation of scientific and rational energy and environmental policies that will give Beijing the confidence to successfully achieve its carbon reduction goal.

There are many studies on the factors that influence CE, but there is still room for improvement. First, the existing studies on the impact of EI on CE are generally conducted at the level of decomposition of the influencing factors of CE. Although the decomposition analysis of the influencing factors of CE can comprehensively measure the contribution of different factors to CE, it cannot clarify the elasticity coefficient of EI on CE. Second, the existing studies on the relationship between EI and CE often do not take into account the potential role of spatial factors and do not reveal the mechanism of EI on CE, leading to specific research gaps in the relevant areas. Finally, existing studies tend to be national or regional in scope, but relatively few studies address the relationship between EI and CE in Beijing. To address the above problems, this study first measures the data of CE for Beijing from 2010 to 2020, and then decomposes the factors affecting CE by constructing the logarithmic mean Divisia index (LMDI) model. Second, the spatial Dubin model (SDM) is used to analyse the spatial spillover effect in the process of EI influencing CE reduction. Finally, the macro-regulatory role of economic development in EI influencing CE is analysed. This study reveals the influence mechanism and characteristics of EI on CE in Beijing, thus providing scientific theoretical support and policy recommendations for Beijing to promote the goal of carbon neutrality by 2050.

The rest of this paper is structured as follows: Section 2 reviews the literature. Section 3 describes the data and methodology. Section 4 contains the analysis and discussion of the results. Finally, Section 5 concludes the whole study and suggests relevant countermeasures.

## 2. Literature Review

In recent years, CE has gained increasing attention in the face of climate deterioration [20,21,22]. Scholars have now conducted fruitful studies of CE around the world [23,24,25]. Moutinho et al. [26] analyse the relevant factors driving CE changes in Europe and find that CE is closely linked to energy consumption and structure. Xu et al. [27] point out that economic performance, population size, and energy structure are the most important factors affecting changes in CE in China, with economic performance having the greatest influence. Wu et al. [28] and Zhang et al. [22] conclude that energy structure in China has a smaller influence on CE, while EI is the main factor affecting changes in CE. Mousavi et al. [29] investigate the change factors of CE in Iran and suggest that the increase in CE is mainly due to rapid economic development and an increase in energy consumption, and the increase in the share of traditional fossil energy in Iran’s energy structure has also contributed to CE. Sumabat et al. [30] examine the influencing factors of CE in the Philippines and find that economic growth and people’s lifestyle are the most important influencing factors of CE. Doganlar et al. [31] highlight a long-term influence of economic growth, energy consumption, and financial development on CE using data for Turkey from 1965–2018. Sun et al. [32] investigate the main driving factors for CE in Belt and Road Initiative countries and identify population, GDP per capita, EI, renewable energy, and urbanization as five key factors. Ertugrul et al. [33] examine the relationship between CE, trade openness, real income, and energy consumption using a sample of the ten largest carbon emitters among emerging economies from 1971–2011.

Some articles have conducted an in-depth analysis of CE at the sectoral level [34,35,36]. Diakoulaki and Mandaraka [37] examine the changes in industry CE in EU countries from 1990 to 2003 and point out that production, EI, fuel mix, and utility mix are the main factors affecting CE. Kopidou and Diakoulaki [38] explore the factors influencing CE from the industrial sectors in four southern European countries and find that EI and economic activities are the most important factors influencing CE variations. Lin and Benjamin [39] suggest that GDP, EI, and carbon intensity are the key factors influencing CE in the transport sector in China. Akbostanci et al. [40] analyse the factors causing changes in CE in 57 manufacturing industries in Turkey and find that changes in EI are the most important factor for changes in CE. Li et al. [41] investigate the main drivers of CE in the agricultural sectors in 18 European countries and conclude that changes in CE are closely related to EI. O’Mahony [42] examines the drivers of CE in 11 final energy consumption sectors in Ireland from 1990 to 2010 and concludes that EI and fossil fuel substitution can offset the increase in emissions due to the scale effect of wealth and population growth. Han et al. [43] analyse the factors affecting CE changes in Chinese agriculture and suggest that the scale effect of agricultural development contributes positively to CE. Lin and Long [44] study CE in China’s chemical industry and show that per capita production, scale of industrial economy, EI, and energy structure affect CE significantly in the chemical industry. In addition to the above influencing factors, industrial structure [45,46], urbanisation [21,47], foreign direct investment [48,49], energy price [50,51], renewable energy consumption [52,53], research and development (R&D) intensity [54], and green technology innovation [55] are also important factors affecting the variations of CE.

In recent years, structural decomposition analysis (SDA) and index decomposition analysis (IDA) have become important tools for analysing the drivers of CE. The core of the SDA method is to convert the change of CE into the resulting force of the changes of different associated driving forces to calculate the contribution of each driving force to the change of CE [56]. Compared to the IDA method, the SDA method has certain advantages in distinguishing between indirect effects (e.g., technical effects) and direct effects, but this method needs to draw on the inputs and outputs of the whole ecosystem, which in turn requires a relatively large amount of data [57]. In contrast, the IDA method, which has relatively lower data requirements and is more flexible in problem formulation, is widely used to analyse the drivers of CE [44,56]. For the IDA method, the logarithmic mean Divisia index (LMDI) model is typical due to its path independence, aggregation consistency and ability to handle zero values [58,59,60,61]. For example, Dong et al. [62] decompose the drivers of CE for 133 countries using the LMDI and find that a reduction in EI effectively curbs CO_2_ emissions, while an increase in GDP significantly increases CE. Hasan and Chongbo [63] conduct a decomposition analysis of the drivers of CE in Bangladesh using the LMDI method and find that government policies, population, and substitution outcomes have a positive impact on reducing CE.

For China, Wang and Yan [64] point out that economic growth is the primary driver of carbon emission growth, while EI is the most critical factor in reducing CO_2_ emissions, and energy structure and industrial structure are not significant inhibitors of CE. Yang et al. [65] examine the influencing factors of CO_2_ emissions in China by the LMDI method and suggest that economic growth is the most significant driver of CE, while energy consumption intensity is the main factor inhibiting CE. Liu et al. [66] analyse the influencing factors of CO_2_ emissions in China’s transport sector based on the LMDI model and find that the capital investment effect is the key factor promoting CO_2_ emissions. Some studies have analysed the influencing factors of CE at the provincial level in China. Liu et al. [67] decompose and analyse the influencing factors of economic development in Sichuan province by the LMDI method and propose that population size and economic development positively contribute to CO_2_ emissions. Gu et al. [68] examine the influencing factors of CE in Shanghai using the LMDI method and highlight that GDP per capita is the primary driver of CE growth in Shanghai, while energy consumption intensity is the main influencing factor of CE reduction. Chen et al. [69] conduct a decomposition analysis of industrial CO_2_ emissions in Dalian and point out that the economic growth effect is the most important factor affecting industrial CO_2_ emissions, while the industrial structure is the most significant influencing factor in reducing CE.

A large body of studies indicate that economic growth is one of the main drivers of CE. However, there is not yet a unified academic consensus on the relationship between EI and CE. Some scholars believe that there is a positive relationship between EI and CE [20,70]. Wang et al. [20] apply an improved patron-driven acquisition (PDA) model to investigate the impact of changes in CE based on Chinese data from 2005–2010 and conclude that reducing EI can effectively mitigate the growth rate of CE. Some researchers point out that EI has no significant impact on CE. Wang and Zhao [21] believe that energy savings from energy efficiency are offset by new energy demand due to energy prices, income and economic effects, leading to an increase in CE. Other scholars believe that the impact of EI on CE reduction is not significant. Wu et al. [71] point out that changes in the share of renewable energy and carbon intensity will be the two most important factors in reducing CE over the next 30 years, while the contributions of industry structure, economic growth, and EI are rather small. The relationship between EI and CE in Beijing cannot be clarified by existing studies. Therefore, analysing the influencing factors of CE and further revealing the relationship between EI and CE in Beijing can enrich and improve research in related fields.

## 3. Data and Methodology

### 3.1. Estimation of Carbon Emissions

According to IPCC [72], CE can be measured by converting the consumption of different energy sources. In this study, 12 main categories of fossil fuels are selected to measure CE. The specific calculation formula is as follows.

(1) $$C=\sum_{i=1}^{12}C_{i}=\sum_{i=1}^{12}E_{i}\times NCV_{i}\times CEF_{i}\times COF_{i}\times \frac{44}{12}$$

where C stands for CE; i stands for energy fuels, including raw coal, cleaned coal, briquettes, coke, gasoline, kerosene, fuel oil, diesel oil, liquefied petroleum gas, refinery gas, other petroleum products, and natural gas; $E_{i}$ represents energy consumption; $NCV_{i}$ is the average low calorific value; $CEF_{i}$ denotes the CE coefficient; $COF_{i}$ is the carbon oxidation factor; 12/44 is the ratio of the molecular weights of carbon and carbon dioxide. Table 1 lists the relevant measured coefficients of CE for each energy source.

### 3.2. Models

There are two main approaches to analysing the factors influencing CE: decomposition analysis and the econometric method based on regression models [73]. The advantage of decomposition analysis is that the contribution of the different factors to CE can be measured comprehensively, and the advantage of panel regression is that the elasticity coefficient of each component to CE can be clarified [74]. Based on the decomposition analysis of the factors influencing CE, this study aims to use econometric methods to investigate the influence of EI on CE.

#### 3.2.1. The Decomposition Method

In this study, the LMDI method is used to investigate the driving factors of CE in Beijing, and the formula is as follows:

(2) $$C=\sum_{i=1}^{5}C_{i}=\sum_{i=1}^{5}\frac{C_{i}}{E_{i}}\times \frac{E_{i}}{Y_{i}}\times \frac{Y_{i}}{Y}\times \frac{Y}{P}\times P=\sum_{i=1}^{5}CI_{i}\times EI_{i}\times IS_{i}\times YP\times P$$

where C is the total amount of CE in Beijing; i denotes the industrial sectors, including the agricultural sector, the manufacturing sector, the construction sector, the transport sector, and the services sector. $C_{i}$ is the CE produced by the i sector; $E_{i}$ represents energy consumption; $Y_{i}$ is the GDP in the i sector; Y is the GDP in Beijing; P represents population. $CI_{i}=C_{i}/E_{i}$ is the carbon intensity expressed in CE per unit of energy consumption. $EI_{i}=E_{i}/Y_{i}$ is the EI expressed as energy consumption per unit of GDP; $IS_{i}=Y_{i}/Y$ is the ratio of the i sector’s GDP to the city’s GDP, reflecting Beijing’s industrial structure. YP=Y/P is the GDP per capita, reflecting the economic development.

Then, Equation (2) can be used to determine the cumulative change in CE in year t compared to the base year:

(3) $$\Delta C=C^{t}-C^{0}=\Delta CI+\Delta EI+\Delta IS+\Delta YP+\Delta P$$

where ΔC is the total effect. ΔCI is the carbon intensity effect, reflecting the contribution of changes in CE per unit of energy to the total change in CE. ΔEI is the EI effect, reflecting the influence of changes in energy use efficiency on the contribution to the total change in CE. ΔIS is the industrial structure effect, reflecting the contribution of adaptation and modernisation of the industrial structure to the overall change in CE. ΔYP is the economic development effect, reflecting the contribution of changes in economic growth to the overall change in CE. ΔP is the population size effect, reflecting the contribution of changes in population size to the overall change in CE. The contribution of each decomposition factor is calculated as follows:

(4) $$\begin{array}{c} \Delta CI=\sum_{i}^{5}\frac{C_{i}^{t}-C_{i}^{0}}{\ln C_{i}^{t}-\ln C_{i}^{0}}\cdot \ln\frac{CI_{i}^{t}}{CI_{i}^{0}};\;\;\Delta EI=\sum_{i}^{5}\frac{C_{i}^{t}-C_{i}^{0}}{\ln C_{i}^{t}-\ln C_{i}^{0}}\cdot \ln\frac{EI_{i}^{t}}{EI_{i}^{0}};\;\\ \Delta IS=\sum_{i}^{5}\frac{C_{i}^{t}-C_{i}^{0}}{\ln C_{i}^{t}-\ln C_{i}^{0}}\cdot \ln\frac{IS_{i}^{t}}{IS_{i}^{0}};\;\Delta YP=\sum_{i}^{5}\frac{C_{i}^{t}-C_{i}^{0}}{\ln C_{i}^{t}-\ln C_{i}^{0}}\cdot \ln\frac{YP_{i}^{t}}{YP_{i}^{0}};\;\\ \Delta P=\sum_{i}^{5}\frac{C_{i}^{t}-C_{i}^{0}}{\ln C_{i}^{t}-\ln C_{i}^{0}}\cdot \ln\frac{P_{i}^{t}}{P_{i}^{0}} \end{array}$$

The change in CE in year t compared to year t−1 is thus calculated as:

(5) $$\Delta C=C^{t}-C^{t-1}=\Delta CI+\Delta EI+\Delta IS+\Delta YP+\Delta P$$

where the contribution of each decomposition factor is expressed as:

(6) $$\begin{array}{c} \Delta CI=\sum_{i}^{5}\frac{C_{i}^{t}-C_{i}^{t-1}}{\ln C_{i}^{t}-\ln C_{i}^{t-1}}\cdot \ln\frac{CI_{i}^{t}}{CI_{i}^{t-1}};\;\;\Delta EI=\sum_{i}^{5}\frac{C_{i}^{t}-C_{i}^{t-1}}{\ln C_{i}^{t}-\ln C_{i}^{t-1}}\cdot \ln\frac{EI_{i}^{t}}{EI_{i}^{t-1}};\;\\ \Delta IS=\sum_{i}^{5}\frac{C_{i}^{t}-C_{i}^{t-1}}{\ln C_{i}^{t}-\ln C_{i}^{t-1}}\cdot \ln\frac{IS_{i}^{t}}{IS_{i}^{t-1}};\;\Delta YP=\sum_{i}^{5}\frac{C_{i}^{t}-C_{i}^{t-1}}{\ln C_{i}^{t}-\ln C_{i}^{t-1}}\cdot \ln\frac{YP_{i}^{t}}{YP_{i}^{t-1}};\;\\ \Delta P=\sum_{i}^{5}\frac{C_{i}^{t}-C_{i}^{t-1}}{\ln C_{i}^{t}-\ln C_{i}^{t-1}}\cdot \ln\frac{P_{i}^{t}}{P_{i}^{t-1}} \end{array}$$

#### 3.2.2. The Econometric Model

To analyse the impact of EI on CE, this study first uses the classical OLS model to construct the following benchmark regression:

(7) $$\begin{array}{l} \ln C_{it}=\alpha_{1}\ln EI_{it}+\alpha_{2}\ln ECO_{it}+\alpha_{3}\ln POP_{it}+\alpha_{4}\ln TEC_{it}\\ \;\;\;+\alpha_{5}\ln STR_{it}+\alpha_{6}\ln OPE_{it}+\mu_{i}+\varepsilon_{it} \end{array}$$

where i represents the municipal district of Beijing, and t represents the year. C is the dependent variable; EI is the central explanatory variable. ECO, POP, TEC, STR, and OPE are the control variables representing the level of economic development, the size of the population, the level of technological innovation, the level of advanced industry, and the degree of openness to the outside world. $\mu_{i}$ is the individual effect and $\varepsilon_{it}$ is the random error.

Tobler [75] points out that everything correlates with each other and that the correlation between things that are close to each other is stronger. Therefore, it is necessary to study the spatial spill over effect of EI on CE. LeSage and Pace [76] constructed the spatial Durbin model (SDM) in 2009, which takes into account not only two spatial transmission mechanisms of the dependent variable and the error term but also the spatial interaction. That is, CE is not only influenced by the EI of this region but also by changes in CE and EI in other regions. Therefore, this study uses the SDM model to examine the spatial spillover effect of EI on CE. The model formula is as follows:

(8) $$\begin{array}{l} \ln C_{it}=\beta_{1}\ln EI_{it}+\beta_{2}\ln ECO_{it}+\beta_{3}\ln POP_{it}+\beta_{4}\ln TEC_{it}\\ \;\;\;+\beta_{5}\ln STR_{it}+\beta_{6}\ln OPE_{it}+\mu_{i}+\nu_{it}\\ \nu_{it}=\lambda W\nu_{it}+\varepsilon_{it}\;\;,\;\;\varepsilon_{it}∼N\left(0,\;\sigma^{2}I\right) \end{array}$$

where $\nu_{it}$ is the error term. W is the spatial weight matrix.

To further investigate the impact mechanism of EI on CE, this study uses economic development as a moderating variable to test the macro-regulatory effect of economic development on energy in the process of CE reduction. Therefore, based on the benchmark model (7), the interaction term between EI and economic development is introduced and the moderating effect of economic development on the impact path of EI on CE is examined. Thus, model (9) can be obtained. In the benchmark model (7) and model (9), if both $\alpha_{1}$ and $\gamma_{1}$ are positive (negative) and $\gamma_{c}$ is positive (negative), economic development has an amplified moderating effect, i.e., the expansion of economic scale amplifies the impact of EI on CE. When both $\alpha_{1}$ and $\gamma_{1}$ are positive (negative), but $\gamma_{c}$ is negative (positive), economic development has a disruptive moderating effect, i.e., the expansion of economic scale weakens the impact of EI on CE.

(9) $$\begin{array}{l} \ln C_{it}=\gamma_{1}\ln EI_{it}+\gamma_{2}\ln ECO_{it}+\gamma_{c}\ln EI_{it}\cdot \ln ECO_{it}+\gamma_{3}\ln POP_{it}\\ \;\;\;+\gamma_{4}\ln TEC_{it}+\gamma_{5}\ln STR_{it}+\gamma_{6}\ln OPE_{it}+\mu_{i}+\varepsilon_{it} \end{array}$$

To further test whether economic development has a nonlinear moderating effect, the following panel threshold model is constructed:

(10) $$\begin{array}{l} \ln C_{it}=\rho_{1}\cdot \left[\ln EI_{it}\times I(s_{it}\leq q)\right]+\rho_{2}\cdot [\ln EI_{it}\times I(s_{it}>q)]+\rho_{3}\ln ECO_{it}\\ \;\;\;+\rho_{4}\ln POP_{it}+\rho_{5}\ln TEC_{it}+\rho_{6}\ln STR_{it}+\rho_{7}\ln OPE_{it}+\mu_{i}+\varepsilon_{it}\; \end{array}$$

where $s_{it}$ is the threshold variable, representing the scale of economic development, q is the threshold parameter, and I(⋅) is the indicator function, which is equal to 1 if the condition is satisfied, and 0 otherwise. When $s_{it}<q$, the marginal effect of EI on CE is $\rho_{1}$, and when $s_{it}>q$, the marginal effect of EI on CE is $\rho_{2}$. Considering that there may be multiple thresholds, this study constructs a panel data model with multiple thresholds as follows:

(11) $$\begin{array}{l} \ln C_{it}=\rho_{c}\cdot \left[\ln EI_{it}\times I(s_{it})\right]+\rho_{3}\ln ECO_{it}+\rho_{4}\ln POP_{it}\\ \;\;\;+\rho_{5}\ln TEC_{it}+\rho_{6}\ln STR_{it}+\rho_{7}\ln OPE_{it}+\mu_{i}+\varepsilon_{it}\; \end{array}$$

### 3.3. Data and Variables

Two main datasets are used in this study: the time series data in the decomposition analysis of the CE drivers by LMDI and the panel data in the analysis of the relationship between EI and CE by econometric models. In the decomposition analysis of the CE drivers in Beijing by LMDI method, the energy consumption data are from China Energy Statistical Yearbook and the process-related data are from Beijing Statistical Yearbook. When measured using panel data, CE (C) is the explanatory variable and EI (EI) is the key explanatory variable. Economic development level (ECO), population size (POP), technological innovation level (TEC), industrial structure upgrading (STR), and openness to the outside world (OPE) are control variables. Additionally, ECO is denoted by the unit GDP of each region, POP is measured by the population density of each region, TEC is calculated by the number of patent applications, STR is measured by the ratio of the GDP of the tertiary industry to the secondary industry, and OPE is expressed by the amount of actual utilized foreign direct investment. The relevant indicator data all come from the China Energy Statistical Yearbook, the Beijing Statistical Yearbook, the Wind Database, and the Carbon Emission Accounts and Datasets (CEADs). Table 2 provides descriptive statistics for the variables.

## 4. Empirical Results

This section is divided into three subsections that describe the experimental results, their interpretation, and the experimental conclusions that can be drawn.

### 4.1. Decomposition Analysis of the Driving Factors for Carbon Emissions

**Figure 2:**
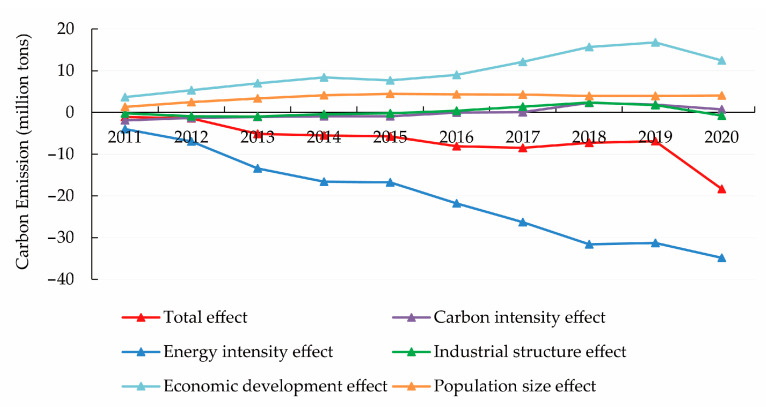
Cumulative contribution of five decomposition factors to carbon emissions.

**Figure 3:**
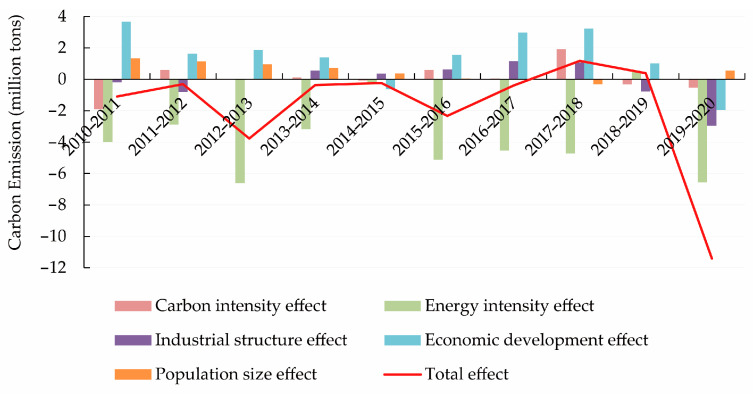
The annual contribution of five decomposition factors to carbon emissions.

From Equations (2)–(6), the cumulative contribution of each decomposition factor of CE (Figure 2) and the annual contribution (Figure 3) can be calculated. A contribution value greater than zero for the LMDI decomposition in additive form indicates that the influencing factor contributes positively to CE. In contrast, a contribution value less than zero indicates that the influencing factor inhibits CE. Specifically, CE decreases by 18.3368 million tonnes from 2010 to 2020, and the total CE shows a significant downward trend. Among them, the impacts of economic development, population size, and carbon intensity are the main drivers for the increase of CE in Beijing. In contrast, the impacts of EI and industrial structure are the main drivers of the decrease of CE.

Among the five decomposition factors, the effect of economic development is the most significant factor contributing to the growth of CE. From the decomposition results of LMDI model, the cumulative contribution of economic development effect to the growth of CE is always positive, and the effect of economic development drives the cumulative increase of CE in Beijing from 2010 to 2020 by 12.4687 million tonnes. In terms of annual contribution, the economic development effect has a weak inhibitory effect on CE in 2014–2015 and 2019–2020, while most other years have a significant promoting effect.

Figure 2 shows that the cumulative contribution of the population scale effect to CE is always positive. From 2010 to 2020, this effect increases CE by 4.0466 million tonnes, which is the second largest after the effect of economic development on CE. In terms of annual contribution, the effect of population size has a weak inhibitory effect in 2016–2017, 2017–2018, and 2018–2019, while it has a more obvious promoting effect in the other years. Overall, among the five decomposition factors, the effect of population size is the second most important factor for the growth of CE in Beijing.

The effect of carbon intensity on CE is relatively weak. Figure 3 shows that carbon intensity has a significant inhibitory effect on CE in 2010–2011, a significant promoting effect in 2017–2018, while the effect on CE is not significant in the other years. Figure 2 shows that the cumulative contribution of the carbon intensity effect to CE has a slightly inhibitory effect before 2016 and then changed to a facilitative effect. Throughout the sample period, carbon intensity has a positive effect on CE and contributes to an increase of 0.7340 million tonnes on CE. Although there is a positive impact on CE, its contribution is much smaller than the effect of economic development and population size.

EI refers to the level of energy consumption per unit of GDP, reflecting the extensive use of energy in economic activities. In the LMDI decomposition results, the EI effect shows a weak contribution to CE in 2018–2019 and a significant inhibitory effect in the other years. Figure 2 shows that the EI effect is always negative in the cumulative contribution to CE. The EI effect contributes to a cumulative reduction of 34.8172 million tonnes in CE in Beijing from 2010 to 2020. The suppression effect of EI on CE is much larger than the promotion effect of economic development, population size, and carbon intensity on CE, which is the main factor for the decrease of CE in Beijing [11].

The influence of the industrial structure on CE is subject to considerable fluctuations. In terms of annual contribution, the impact of industrial structure has a dampening effect on CE in 2010–2011, 2011–2012, and 2012–2013, while it has a strengthening effect on CE in each year of the 2014–2018 period, and again has a dampening effect on CE in 2018–2019 and 2019–2020. In terms of cumulative contribution, the effect of industrial structure has a dampening effect on CE, and the effect of industrial structure contributes to a decrease of CE by 0.7690 million tonnes during 2010–2020. Compared to the EI effect, the impact of the industrial structure effect on the reduction of CE is relatively small.

The breakdown of the CE influencing factors shows that the EI effect is the key factor for the decline of CE in Beijing. Therefore, this study further analyses the EI effect from the perspective of the five main CE sectors in Beijing. Table 3 shows the cumulative contribution of EI effects to CE in each sector. The cumulative contribution of EI effect to the reduction of CE in the manufacturing sector continues to increase from 2010–2018, followed by a decreasing trend from 2019–2020. Among the sectors, the contribution of EI effect to the reduction of CE in this sector is the highest, with a total emission reduction of 21.4063 million tonnes during the study period, accounting for 61.48% of the total emission reduction in all sectors.

In the transport sector, the contribution of the EI effect is relatively small from 2010 to 2015 and increases significantly after 2015. Overall, the cumulative emission reduction of the EI effect in the transport sector from 2010 to 2020 is 7.1460 million tonnes, which accounts for 20.52% of the total EI reduction effect. Additionally, the EI effect of the services sector and the construction sector varies only slightly, reaching emission reductions of 3.0381 million tonnes and 2.2916 million tonnes, representing 8.73% and 6.58%, respectively, during the study period. Finally, the agricultural sector has the smallest EI effect, reducing CE by only 0.9352 million tonnes over the entire study period, which corresponds to a contribution to an emission reduction of 2.69%.

Table 4 shows the measured results of the annual contribution of the EI effect to CE in each sector. It can be seen that prior to 2017, the manufacturing sector had the highest contribution of the EI effect to the reduction of CE each year. However, from 2017 to 2018, the contribution of the manufacturing sector to the reduction of CE is gradually overtaken by the transport and services sectors. The contribution of the manufacturing sector to the reduction of CE in 2020 is only higher than that of the agricultural sector. It lags behind the construction, transport, and services sectors. This reflects the fact that with the reform of industrialisation in Beijing, energy efficiency has greatly improved, and the problem of large amounts of CE caused by coarse industrial production has been significantly improved.

After 2015, the annual contribution of the transport sector to the reduction of CE has increased significantly (except 2018–2019). In particular, the contribution of the transport sector to the reduction of CE in 2019–2020 far exceeds the contribution of the other four sectors. One possible reason is that new technologies are enabling the transport sector to gradually move away from reliance on fossil fuels such as petrol and diesel, which contributes significantly to the reduction of CE in this sector. The services sector is similar to the construction sector in that both sectors show an increasing trend in their annual contribution to the reduction of CE over time. The EI of the agricultural sector plays a relatively minor role in reducing CE and the sector’s contribution to reducing CE shows a decreasing development characteristic.

### 4.2. Analysis of the Spatial Spill over Effects

**Figure 4:**
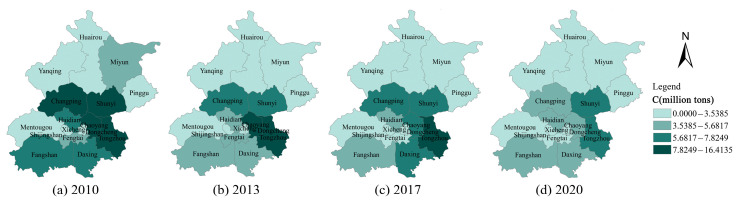
Development trend of carbon emissions in Beijing’s municipalities from 2010 to 2020.

**Figure 5:**
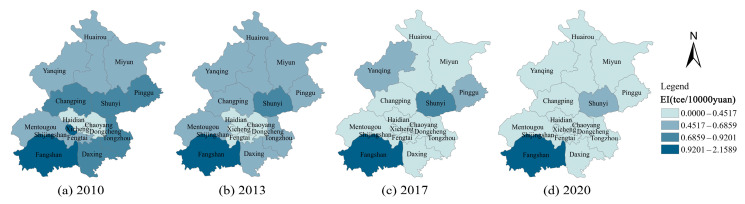
Development trend of energy intensity in Beijing’s municipalities from 2010 to 2020.

Figure 4 shows the regional distribution and development trend of CE in the different municipalities of Beijing. It can be found that the values of CE are generally low in the neighbouring areas of Yanqing, Huairou, Miyun, and Pinggu, which are located in the north. In contrast, the levels of CE are generally high in Changping, Shunyi, Tongzhou, Haidian, Chaoyang, Fangshan, and Daxing, which are located in the south-central region. Over time, CE declines significantly in the south-central region, but remains high compared to the northern region. Figure 5 shows the regional distribution characteristics and the development of EI in Beijing. It can be seen that Dongcheng, Xicheng, Haidian, and Chaoyang, which are located in the central region, have lower EI in 2010, while the surrounding areas generally have higher EI. Over time, the EI of each municipality decreases significantly, and in 2020, only Fangshan district had a higher EI, while the other districts have a lower EI. Both CE and EI have similar distribution characteristics in neighbouring areas, suggesting that there may be some spatial correlation between the municipalities.

In this study, Moran’s I index [77] is also used to test the spatial correlation of CE and EI. From Figure 4 and Figure 5, it can be seen that CE and EI have some similarities between neighbouring regions. Therefore, the neighbouring weight matrix (w1) is used to measure Moran’s index. Table 5 shows the results of measuring the Moran’s index of CE and EI. For CE, the Moran’s index varies between 0.2070 and 0.2480 from 2010 to 2020. The Moran’s index is positive and all at a significance level of 0.05, which means that CE is positively correlated across regions.

Similarly, Moran’s I index for EI fluctuates between 0.0500 and 0.1980 with positive values. It is within the significance level of 0.1 in most years, which means that EI has spatial autocorrelation. Therefore, it is necessary to consider spatial factors when analysing the relationship between EI and CE in this case.

Table 6 shows the regression results of EI on CE in Beijing. Column (1) corresponds to the regression results of the benchmark model. The regression coefficient of lnEI is 0.1470 and at the significance level of 0.1, showing a positive correlation between EI and CE. The OLS model reflects the most basic correlation between EI and CE, but ignores the spatial factor, so its regression results may differ somewhat. For this reason, the SDM model is used to examine the influence of EI on CE. Column (2) shows that the coefficient of EI on CE is positive and significant, which means that a 1% reduction in EI can effectively contribute to a 0.2346% reduction in CE. There is also a positive correlation between EI and CE, when a spatial correlation is taken into account. To check the robustness and reliability of the results, the results are further investigated by replacing the spatial weights and spatial models. Columns (3)–(4) show the regression results using the geographical weight matrix and the economic distance weight matrix, respectively. Columns (5)–(6) are the regression results under the two spatial regression models of the spatial autoregressive model (SAR) and the spatial error model (SEM). The results do not change fundamentally regardless of the change in the weight matrix or the spatial model, which suggests that the conclusion of a positive correlation between EI and CE is reliable. That is, reducing EI can effectively mitigate CE.

Furthermore, this study divides the effects of EI on CE into direct, indirect, and total effects, according to LeSage and Pace [76]. Table 7 shows that in the short term, each 1% reduction in EI in one area results in a 0.3416% reduction in CE, while CE decreases by 0.9530% in neighbouring areas. The indirect effect accounts for 73.61% of the total effect, reflecting the significant spatial spillover effect of EI on CE. In the long run, a 1% decrease in EI leads to a 0.3687% decrease in the local CE and a 1.1909% decrease in neighbouring regions, so the indirect effect accounts for 76.36% of the total effect. The spatial spillover effect of EI is also pronounced in the long run. In terms of the overall effect, in the short run, a 1% decrease in EI leads to a 1.2947% decrease in CE. In the long run, however, a 1% decrease in EI leads to a 1.5596% decrease in CE, showing that the long-run effect of a decrease in EI is larger than the short-run effect.

### 4.3. Influence Mechanism Analysis

To achieve Beijing’s goal of carbon neutrality by 2050, energy consumption, ecological development, and CE are the main issues considered by policymakers. When examining the relationship between EI and CE, it is, therefore, necessary to consider the role that economic factors play in this process. For both countries and companies, technological innovations are the most important way to reduce EI [19]. Green technological innovations not only reduce EI and thus lower CE by improving production efficiency [78], but also help develop new energy sources and change the industrial structure to reduce EI and, thus, lower CE [79,80,81]. In this process, developed economies can provide continuous capital investment for R&D and innovation, which guarantees the continuous promotion of green technological innovations, thus enabling continuous reduction of EI and CE. At the same time, developed economies place higher demands on the green environment. They are more willing to reduce pollution from energy-intensive industries, so promoting CE reduction is easier in developed economies. From this perspective, macroeconomics has a positive regulatory role in EI on CE reduction.

**Figure 6:**
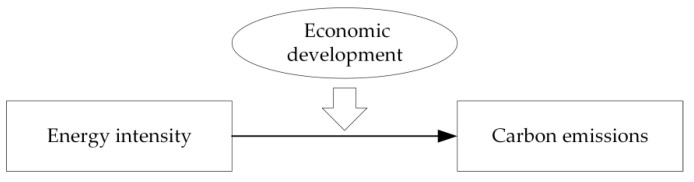
Moderating effects of economic development on energy intensity and carbon emissions.

To verify the above conjecture, the moderating effect of economic development in the process of EI is tested at CE using Equation (9). Table 8 shows the regression results, indicating that the regression coefficient of the interaction term of EI and economic development is significantly positive, and the regression coefficient of EI to CE remains positive. Therefore, it can be concluded that economic factors have an enhanced moderating effect on the process of EI on CE (Figure 6), i.e., a developed economic level can enhance the promoting effect of EI on the reduction of CE.

The preceding analysis shows that economic factors contribute to improving the impact of EI on CE. To further identify the potential non-linear moderating effect of economic factors, Equation (10) is used to analyse the threshold effect. Table 8 shows the test results, suggesting that economic development exceeds the simple threshold at a significance level of 0.10, and the corresponding threshold is 2.7868. The double and triple thresholds are not significant, indicating that economic factors have a non-linear effect on the simple threshold to reduce CE by EI. When the economic development level is below the simple threshold, the regression coefficient of EI is 0.2152 at a significance level of 0.10, indicating that a 1% reduction in EI leads to a 0.2152% reduction in CE. When the economic level is above the critical value, a 1% reduction in EI leads to a 0.3003% reduction in CE. Therefore, the reduction of CE by EI is more effective in regions with a higher level of economic development. From Section 4.1 and Section 4.2, economic development has a facilitating effect on CE. However, considering that the inhibiting effect of EI on CE is much higher than the promoting effect of economic development, it can be concluded that promoting economic development and lowering EI is an effective way to reduce CE.

Analysis of regional economic development level data shows that only the economic development level of Xicheng District exceeds the threshold of 2.7868 from 2010 to 2012. The economy of Dongcheng District develops rapidly and exceeds the critical value of 2.7868 in 2013, and the economic development level of Haidian and Chaoyang districts exceeds the economic threshold in 2017 and 2018. Among the 16 municipalities of Beijing in 2020, only Dongcheng, Xicheng, Chaoyang, and Haidian districts have economic development levels above the threshold. In these districts, CE can be significantly reduced by reducing EI. In the remaining 12 municipalities, CE can still be reduced by lowering EI, but the lowering effect of EI is not maximised. Therefore, in most areas of Beijing, the regional economy needs to be strongly developed to maximise the effect of lowering CE.

## 5. Discussion and Conclusions

### 5.1. Discussion

This study shows that economic expansion is the main factor contributing to the growth of CE in Beijing [64]. The expansion of the economy is often accompanied by increased energy consumption and high demand for industrial products, which leads to a continuous increase in carbon dioxide emissions. However, the inhibiting effect of reducing EI on CE is much greater than the promoting effect of economic development and population growth on CE. Therefore, reducing EI is key to promoting CE reduction and achieving carbon neutrality in Beijing [65]. Broken down by sector, the manufacturing sector has reduced 21.4063 million tonnes CE through EI reduction in the last ten years, making it the most important sector in reducing CE through EI reduction. Due to the development of new energy-powered vehicles and the impact of the epidemic, the transport sector in Beijing has achieved a significant reduction of CE in recent years, gradually making it the core sector of emission reduction. Therefore, new energy vehicles are key to promoting CE reduction in Beijing’s next phase.

EI has a spatial spillover effect on CE, both in the short and long term. This means that reducing EI in one region not only reduces CE in that region, but also effectively reduces CE in neighbouring regions. This corresponds to the actual situation. Since all regions of Beijing are interconnected in terms of economic development and industrial construction, the process of improving energy efficiency and reducing energy intensity in one region inevitably affects the economic development and CE of surrounding regions. It should be noted that reducing EI has a significantly higher effect on emission reduction in the long term. Therefore, the effect of EI on CE is continuous, i.e., a reduction in EI can sustainably promote a reduction in CE.

Economic development has a positive moderating effect in the process of EI on CE, and this moderating effect has a non-linear characteristic with a threshold value. Developed economies can provide continuous capital investment for R&D and innovation, which guarantees the continuous promotion of green technological innovations, thus enabling a continuous reduction of EI and CE. In regions whose economic scale is above the threshold, the reduction of EI has a greater impact on the reduction of CE. Only Dongcheng, Xicheng, Haidian, and Chaoyang districts in Beijing have exceeded this economic threshold, while the other regions are generally below it. Beijing thus needs to vigorously develop the regional economy to maximise the impact of CE.

This study has two possible limitations. First, Beijing borders Hebei province and Tianjin city, and Beijing’s carbon dioxide emissions are closely linked to both regions. This study focuses on the impact of a reduction in EI on CE in Beijing but does not consider the impact of Hebei and Tianjin. The surrounding areas of Beijing can be included in future studies to thoroughly investigate the impact of energy intensity on CE. Second, this study uses 2010 to 2020 as the study period due to data limitations. However, due to the adjustment of energy and environmental policies in recent years, there are significant differences between past development patterns and the current reality. Therefore, the limited study period may not reflect the long-term impacts.

### 5.2. Conclusions and Policy Implications

This study measures CE in Beijing from 2010 to 2020 and analyses the factors influencing CE by applying the LMDI decomposition method. Considering the potential influence of spatial factors on the process of EI affecting CE, the spatial spillover effect on CE is investigated using the SDM model. Finally, this study investigates the mechanism of the influence of EI on CE. The main findings of this study are as follows:

First, economic development is the main factor influencing the growth of CE in Beijing. Therefore, Beijing needs to accelerate the transformation of economic growth to ensure synergistic development of the economy and the environment. Second, reducing EI is the key to curbing the growth of CE. Therefore, low-carbon and energy-saving technologies should be developed, and the efficiency of energy use should be vigorously improved. At the same time, industrialisation reform should continue, and support for new energy vehicles should be strengthened to ensure the gradual realisation of Beijing’s goal of carbon neutrality. Third, given the spatial spillover effect of EI on CE, the government should coordinate the development of production and construction in each region. Policy formulation should take into account the development characteristics of surrounding areas while actively promoting coordination and collaboration among regions to achieve synergistic development of regional energy conservation and emission reduction. Finally, economic development has a positive regulating effect on the effect of EI reduction on CE and has a threshold character. At present, economic development in most areas of Beijing is still below the threshold, and the CE reduction effect of EI is not maximised. Therefore, all regions of Beijing need to further promote economic development and strive to optimise the CE reduction effect of EI to ensure Beijing’s goal of carbon neutrality.

## Figures and Tables

**Table 1:** Energy conversion factors and carbon emission coefficients.

Energy Type	NCV (kJ/kg, m^3^)	CEF (kg/GJ)	COF	CO_2_ Emission Factor (kg/kg, m^3^)
Raw Coal	20,908	26.37	0.94	1.9003
Cleaned Coal	26,344	25.41	0.98	2.4044
Briquettes	20,908	33.56	0.9	2.3183
Coke	28,435	29.42	0.93	2.8604
Gasoline	43,070	18.90	0.98	2.9251
Kerosene	43,070	19.60	0.98	3.0179
Diesel Oil	42,652	20.20	0.98	3.0959
Fuel Oil	41,816	21.10	0.98	3.1705
Liquefied Petroleum Gas	50,179	17.20	0.98	3.1013
Refinery Gas	45,998	18.20	0.98	3.0082
Other Petroleum Products	41,816	20.00	0.98	3.0052
Natural Gas	38,931	15.32	0.99	2.165

**Table 2:** Statistical description of the variables.

Variable	Variable Declaration	Mean	S.D.	Min	Max
C	Carbon emissions	4.1772	3.2368	0.4504	16.4133
EI	Energy intensity	0.5070	0.3997	0.0717	2.5358
ECO	Economic level	9.4305	8.4998	2.1347	45.7600
POP	Population size	5198.0800	7265.4570	157.0000	25,787.0000
TEC	Technology innovation	9741.3130	15,999.3000	41.0000	95,140.0000
STR	Industrial structure	4.8740	6.7654	0.5018	39.4860
OPE	Foreign direct investment	74,246.9100	168,205.0000	100.0000	1,161,083.0000

**Table 3:** Cumulative contribution of energy intensity impacts of different sectors on carbon emissions.

Year	Total	Agricultural Sector	Manufacturing Sector	Construction Sector	Transport Sector	Services Sector
2011	−3.9866	−0.1326	−2.2607	−0.5006	−0.9087	−0.1841
2012	−6.9693	−0.3201	−4.8342	−0.8725	−0.6462	−0.2963
2013	−13.4180	−0.4522	−10.6408	−1.2674	−0.4512	−0.6064
2014	−16.5955	−0.5422	−13.0116	−1.3233	−0.9756	−0.7428
2015	−16.7543	−0.5376	−13.5302	−1.3913	−0.7250	−0.5701
2016	−21.8109	−0.6199	−17.6826	−1.6281	−1.0958	−0.7845
2017	−26.3147	−0.7786	−20.0379	−1.7720	−2.6203	−1.1058
2018	−31.6305	−0.9436	−22.0394	−1.9362	−4.0165	−2.6948
2019	−31.2980	−0.9374	−21.9902	−2.0865	−3.3859	−2.8979
2020	−34.8172	−0.9352	−21.4063	−2.2916	−7.1460	−3.0381

**Table 4:** The annual contribution of energy intensity of different sectors to carbon emissions.

Year	Total	Agricultural Sector	Manufacturing Sector	Construction Sector	Transport Sector	Services Sector
2010–2011	−3.9866	−0.1326	−2.2607	−0.5006	−0.9087	−0.1841
2011–2012	−2.8818	−0.1901	−2.4889	−0.3732	0.2804	−0.1101
2012–2013	−6.6117	−0.1348	−5.9625	−0.4142	0.2207	−0.3210
2013–2014	−3.1770	−0.1080	−2.3464	−0.0339	−0.5449	−0.1439
2014–2015	−0.2493	−0.0285	−0.6582	−0.0537	0.2873	0.2038
2015–2016	−5.1235	−0.1095	−4.1827	−0.1853	−0.3915	−0.2544
2016–2017	−4.5331	−0.1876	−2.1919	−0.1004	−1.6905	−0.3628
2017–2018	−4.7154	−0.1708	−1.1599	−0.1553	−1.5027	−1.7266
2018–2019	0.4854	−0.0241	0.0848	−0.1545	0.8100	−0.2307
2019–2020	−6.5568	−0.0098	−0.2858	−0.3328	−5.4760	−0.4525

**Table 5:** Spatial correlation test of carbon emissions and energy intensity.

Year	CE				EI			
	I	sd (I)	z	*p*	I	sd (I)	z	*p*
2010	0.2090	0.1290	2.1480	0.0160	0.0500	0.1220	0.9620	0.1680
2011	0.2090	0.1270	2.1610	0.0150	0.0870	0.1080	1.4260	0.0770
2012	0.2080	0.1270	2.1670	0.0150	0.0990	0.1080	1.5280	0.0630
2013	0.2070	0.1260	2.1700	0.0150	0.1720	0.1090	2.1920	0.0140
2014	0.2160	0.1280	2.2170	0.0130	0.1790	0.1100	2.2310	0.0130
2015	0.2220	0.1290	2.2400	0.0130	0.1790	0.1110	2.2070	0.0140
2016	0.2340	0.1310	2.2980	0.0110	0.1980	0.1170	2.2550	0.0120
2017	0.2410	0.1320	2.3350	0.0100	0.1820	0.1180	2.1100	0.0170
2018	0.2480	0.1330	2.3720	0.0090	0.1370	0.1170	1.7440	0.0410
2019	0.2390	0.1340	2.2810	0.0110	0.1380	0.1160	1.7740	0.0380
2020	0.2370	0.1350	2.2490	0.0120	0.1220	0.1040	1.8100	0.0350

**Table 6:** The spatial spillover effect of energy intensity on carbon emissions.

	(1)	(2)	(3)	(4)	(5)	(6)
lnEI	0.1470 ***	0.2346 ***	0.0471 *	0.1398 ***	0.0872 ***	0.1222 ***
	(0.0389)	(0.0354)	(0.0280)	(0.0246)	(0.0299)	(0.0361)
lnECO	−0.3885 ***	0.0515	−0.0019	−0.1964 ***	−0.0518	−0.2281 ***
	(0.0712)	(0.0678)	(0.0575)	(0.0469)	(0.0645)	(0.0834)
lnPOP	0.0128	0.5597 ***	0.2917 ***	0.2902 ***	0.3062 ***	0.2540 *
	(0.1335)	(0.1041)	(0.0948)	(0.0865)	(0.1051)	(0.1412)
lnTEC	−0.0523 *	0.0124	−0.0730 ***	0.0077	−0.0655 ***	−0.0801 ***
	(0.0267)	(0.019)	(0.0185)	(0.0174)	(0.0201)	(0.0241)
lnSTR	0.0198	0.1413 ***	−0.0103	0.0873 ***	0.0014	−0.0600
	(0.0392)	(0.032)	(0.0272)	(0.0252)	(0.0057)	(0.0435)
lnOPE	0.0074	−0.0022	0.0037	−0.0024	−0.0189	−0.0023
	(0.0075)	(0.0047)	(0.0052)	(0.0048)	(0.0298)	(0.0064)
ρ		0.7239 ***	0.7216 ***	0.7431 ***	0.5847 ***	
		(0.0605)	(0.0552)	(0.0498)	(0.0624)	
λ						0.5233 ***
						(0.1145)
n	176	176	176	176	176	176
R^2^	0.8603	0.8534	0.8842	0.8536	0.8793	0.8517
AIC	−394.4919	−279.7437	−484.4979	−499.8542	−452.1035	−416.9191
BIC	−372.2985	−244.8684	−459.1340	−474.4903	−426.7397	−391.5553

Note: * and *** indicate that the regression coefficients are statistically significant at the 10% and 1% levels, respectively.

**Table 7:** The direct and indirect effects of energy intensity on carbon emissions.

	Short-Term			Long-Term		
	Direct	Indirect	Total	Direct	Indirect	Total
lnEI	0.3416 ***	0.9530 ***	1.2947 ***	0.3687 ***	1.1909 ***	1.5596 ***
	(0.0619)	(0.3411)	(0.3961)	(0.0739)	(0.4890)	(0.5563)
lnECO	0.0307	−0.2252	−0.1945	0.0265	−0.2613	−0.2349
	(0.0770)	(0.3249)	(0.3728)	(0.0829)	(0.4053)	(0.4621)
lnPOP	0.7203 ***	1.3587 *	2.0789 **	0.7642 ***	1.7477	2.5119 **
	(0.1504)	(0.7821)	(0.8993)	(0.1756)	(1.0989)	(1.2460)
lnTEC	0.0284	0.1450	0.1734	0.0322	0.1779	0.2101
	(0.0254)	(0.1238)	(0.1432)	(0.0285)	(0.1609)	(0.1840)
lnSTR	0.1618 ***	0.1727	0.3344 **	0.1686 ***	0.2336	0.4023 *
	(0.0366)	(0.1440)	(0.1665)	(0.0399)	(0.1922)	(0.2196)
lnOPE	0.0000	0.0203	0.0203	0.0004	0.0237	0.0241
	(0.0070)	(0.0293)	(0.0349)	(0.0076)	(0.0365)	(0.0429)

Note: *, **, and *** indicate that the regression coefficients are statistically significant at the 10%, 5%, and 1% levels, respectively.

**Table 8:** The test for the moderating effect and the threshold effect of economic development.

Moderating Effect Test		Threshold Effect Test				
lnEI	0.1481 ***	*p* value				
	(0.0376)		Single	0.0867	lnEI * I (Th < q)	0.2152 ***
lnECO	−0.2912 ***					(0.0350)
	(0.0743)		Double	0.1767	lnEI * I (Th ≥ q)	0.3003 ***
lnEI·lnECO	0.0754 ***					(0.0354)
	(0.0218)		Triple	0.1800		
lnPOP	−0.1475				lnPOP	0.2264 *
	(0.1370)					(0.1157)
lnTEC	−0.0837 ***	Threshold	q1	2.7868	lnTEC	−0.1376 ***
	(0.0273)					(0.0194)
lnSTR	0.0181		q2	3.3539	lnSTR	−0.0765 **
	(0.0379)					(0.0368)
lnOPE	0.0112		q3	1.7495	lnOPE	0.0146 *
	(0.0073)					(0.0076)

Note: *, **, and *** indicate that the coefficients are statistically significant at the 10, 5, and 1% levels, respectively.

## Data Availability

Not applicable.
